# Mitochondrial iron transport and homeostasis in plants

**DOI:** 10.3389/fpls.2013.00348

**Published:** 2013-09-06

**Authors:** Anshika Jain, Erin L. Connolly

**Affiliations:** Department of Biological Sciences, University of South CarolinaColumbia, SC, USA

**Keywords:** iron, mitochondria, plant, iron transporter, frataxin, ferritin

## Abstract

Iron (Fe) is an essential nutrient for plants and although the mechanisms controlling iron uptake from the soil are relatively well understood, comparatively little is known about subcellular trafficking of iron in plant cells. Mitochondria represent a significant iron sink within cells, as iron is required for the proper functioning of respiratory chain protein complexes. Mitochondria are a site of Fe–S cluster synthesis, and possibly heme synthesis as well. Here we review recent insights into the molecular mechanisms controlling mitochondrial iron transport and homeostasis. We focus on the recent identification of a mitochondrial iron uptake transporter in rice and a possible role for metalloreductases in iron uptake by mitochondria. In addition, we highlight recent advances in mitochondrial iron homeostasis with an emphasis on the roles of frataxin and ferritin in iron trafficking and storage within mitochondria.

## INTRODUCTION

Iron is an essential micronutrient for virtually all organisms, including plants. Indeed, photosynthetic organisms are distinguished by the high iron requirement of photosynthetic complexes. Although iron is generally quite abundant in the soil, it has a low bioavailability in aerobic environments at neutral to basic pH and as a result, approximately 30% of the world’s soils are considered iron-limiting for plant growth. Iron deficiency represents an enormous problem in human populations as well, with approximately two billion people afflicted (Wu et al., 2002). Plant foods (especially staples like rice, maize, and wheat) tend to be poor sources of dietary iron and thus significant interest surrounds efforts to develop crop varieties with elevated levels of bioavailable iron.

Despite its importance, iron can be toxic when it accumulates to high levels within cells. It catalyzes the formation of hydroxyl radicals that can damage cellular components like DNA and proteins (Halliwell et al., 1992). Thus iron metabolism is carefully regulated to ensure adequate supply of iron while avoiding toxicity associated with its over-accumulation. Organelles like chloroplasts and mitochondria are thought to play a central role in the cellular iron economy of a plant cell. This is because iron serves as an essential cofactor for many enzymes involved in the electron transport chain in mitochondria and in the photosynthetic complexes found in chloroplasts. Indeed, recent work has shown that iron deficiency results in significant changes in the structure and function of mitochondria (Vigani et al., 2009; Vigani and Zocchi, 2009). PS1–LHC1 supercomplexes also exhibit structural and functional alterations under iron limited conditions (Yadavalli et al., 2012). Thus, iron deficiency affects respiratory and photosynthetic output. However, excessive iron exacerbates the generation of reactive oxygen species (ROS) in these redox centers, which can have exceedingly deleterious effects on cells. Thus, mitochondrial and chloroplast iron metabolism are of particular importance to cellular iron homeostasis (Nouet et al., 2011; Vigani et al., 2013).

The chloroplasts and mitochondria are unique organelles in that they are thought to have evolved via endosymbiosis. As a result, both organelles are surrounded by two membranes; the outer membrane resembles eukaryotic membranes while the inner resembles prokaryotic membranes. Thus, it follows that these two organelles may utilize prokaryotic and/or eukaryotic type iron transport systems (Shimoni-Shor et al., 2010). It is usually assumed that Fe may pass freely across the outer membrane of both organelles via porins. It is also important to note that there is little known about the speciation of cytosolic Fe although it is assumed that there is very little free Fe present in the cytosol (Hider and Kong, 2013). Thus, the Fe species that are available for transport into subcellular compartments are unclear at this time.

**FIGURE 1:**
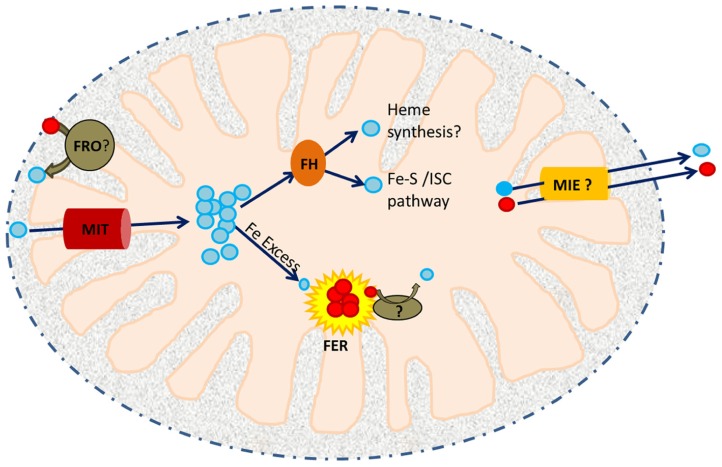
**A working model of iron trafficking and utilization in plant mitochondria.** Cytosolic Fe^3^^+^ (red circles) may be reduced to Fe^2^^+^ (blue circles) by a member of the ferric reductase oxidase (FRO) family within the inter-membrane space (IMS). Ferrous iron is then translocated across the inner membrane by MIT. In the mitochondrial matrix, it is received by an iron chaperone, frataxin (FH). FH distributes this Fe to ISC assembly proteins and possibly to the heme biosynthetic machinery. Excess Fe^2^^+^ is stored in FER4. Iron released from FER4 upon Fe deficiency may require the activity of another reductase prior to its utilization/remobilization. Mitochondrial iron exporters (MIEs) are postulated to function in mitochondrial iron export for delivery of iron to CIA.

A variety of proteins are known to be involved in the maintenance of mitochondrial iron homeostasis (**Figure 1**). The recent discovery and characterization of rice MIT (mitochondrial iron transporter), which is involved in iron uptake by mitochondria, and the mitochondrial iron chaperone, frataxin (FH) has demonstrated the significance of mitochondrial iron uptake and trafficking/distribution to plant growth and development (Busi et al., 2006; Bashir et al., 2011b; Maliandi et al., 2011; Vigani, 2012). Other key players responsible for maintaining mitochondrial iron homeostasis are the iron–sulfur cluster (ISC) synthesis machinery, which accepts iron from FH and mediates the synthesis of Fe–S clusters to serve as cofactors for various proteins in the mitochondria and cytosol (Lill et al., 2012). It is thought that mitochondria also contain the iron storage protein ferritin, which serves to store iron and protect against Fe-catalyzed ROS production (Briat et al., 2010). Mitochondria also supply iron and sulfur to the cytoplasmic iron–sulfur cluster assembly machinery (CIA; Balk and Pilon, 2011). While the sulfur scaffold is likely exported to the cytosol via an ABC transporter, ATM3 (Bernard et al., 2009), putative transporters required for iron efflux are still unknown. The recent discovery of a mitochondrial iron exporter (MIE) in mice (ATP-binding cassette B8, ABCB8) and its role in CIA-mediated Fe–S synthesis has provided new insight into understanding of intracellular iron homeostasis (Ichikawa et al., 2012) and may facilitate the identification of MIE proteins in other species. Finally, a recent report showed that the YSL4 and YSL6 (yellow stripe-like) transporters are involved in iron release from chloroplasts of *Arabidopsis*, suggesting a possible role for other members of the YSL family in mitochondrial iron efflux (Divol et al., 2013). In this review, we discuss in detail the roles of the iron transporter (MIT), metalloreductases, FH and ferritin in mitochondrial iron homeostasis.

## MITOCHONDRIAL IRON TRANSPORTERS

Mitochondrial iron transporters are conserved proteins that belong to the mitochondrial carrier family (MCF; Wiesenberger et al., 1991; Metzendorf et al., 2009; Bashir et al., 2011a, b). This family consists of small (~30 kDa) proteins that localize to the mitochondrial inner membrane and are involved in solute transport (e.g., keto acids, nucleotides, amino acids, etc.) into the mitochondrial matrix (Kunji and Robinson, 2006). MCF proteins were first characterized in yeast and their crystal structure shows the presence of a tripartite structure with a total of six transmembrane helices. Amino acid residues responsible for substrate recognition are found in helices II, IV and VI.

The first set of mitochondrial iron transporters (MRS3 and MRS4 for mitochondrial RNA splicing) were discovered in yeast as multicopy suppressors of the *mrs2 *phenotype (Waldherr et al., 1993) and subsequently shown to function in Fe import (Foury and Roganti, 2002). Mitochondrial iron transporters (named Mitoferrins) have since been identified and characterized in zebrafish, humans, and Drosophila (Shaw et al., 2006; Metzendorf et al., 2009; Paradkar et al., 2009). Later, a mitochondrial iron transporter was identified in rice and named MIT (Bashir et al., 2011b). Similar to zebrafish mitoferrin, rice *MIT* is able to rescue the poor growth phenotype of the yeast *mrs3/4* mutant under iron deficiency, indicating the functional similarity of these proteins. *MIT* is an essential gene as the *mit* knockout mutant shows an embryo lethal phenotype. *mit* knockdown mutants exhibit a slow growth phenotype, reduced chlorophyll concentration and poor seed yield. In addition, these mutants show reduced mitochondrial iron concentration while total iron concentration is elevated, indicating that iron is mislocalized in *mit* loss-of-function lines (Bashir et al., 2011b). In the absence of *MIT*, the gene encoding vacuolar iron transporter1 (*VIT1*) is upregulated, suggesting that excess cytosolic iron may be directed toward vacuoles. MIT plays an important role in seed development and its expression level is positively regulated by iron availability. *MIT *is expressed throughout development, consistent with the idea that it is essential for mitochondrial iron metabolism.

Previous studies conducted in yeast and mammals have demonstrated an adverse effect of loss of mitochondrial iron transport on heme and Fe–S cluster synthesis (Zhang et al., 2005; Shaw et al., 2006; Zhang et al., 2006). In rice, partial loss of MIT results in decreased total and mitochondrial aconitase activity, indicating that the effect on Fe–S cluster synthesis affects not only mitochondrial Fe–S proteins but also cytosolic Fe–S cluster proteins. However, the role of MIT in heme synthesis has yet to be determined. Interestingly, the fact that *mit* loss-of-function lines show altered chlorophyll concentration and altered ferritin expression supports the idea of cross-talk between mitochondrial and chloroplastic iron homeostasis.

Yeast MRS3/4 are thought to serve as high affinity ferrous ion transporters which are essential in the absence of other low affinity mitochondrial iron transporters (Froschauer et al., 2009). Thus, low affinity iron uptake systems may be present at the plant mitochondrial membrane. The recent discovery of siderophore (2,5-DHBA)-mediated iron delivery to mammalian mitochondria has introduced the possibility of such an alternative pathway for iron transport into the mitochondria (Devireddy et al., 2010). 2,5-DHBA is synthesized by a short chain dehydrogenase/reductase family member (BDH2). BLAST searches of the *Arabidopsis* and rice genomes indicate that these genomes code for 3 and 13 BDH2 homologs respectively. Characterization of these homologs may give interesting insights into the mitochondrial iron trafficking pathways in plants.

## PUTATIVE MITOCHONDRIAL FERRIC REDUCTASES

The recent characterization of *Arabidopsis* FRO7 (ferric reductase oxidase 7), a chloroplast-localized member of the FRO family, demonstrated that this protein functions to reduce ferric iron to ferrous iron at the surface of the chloroplast for subsequent uptake into the organelle (Jeong et al., 2008). A similar hypothesis has been suggested for mitochondria based on the predicted localization of FRO3 and FRO8 to mitochondria (Jeong and Connolly, 2009). Whereas *FRO3-GUS* promoter lines show expression throughout seedlings with highest expression in vasculature, expression of *FRO8* is restricted to shoots during senescence (Mukherjee et al., 2006; Jeong and Connolly, 2009). This suggests that the two mitochondrial FROs may be involved in reducing Fe^3^^+^ at different stages of development. *FRO3* has been widely used as an iron deficiency marker (Mukherjee et al., 2006). Nevertheless, the exact role of *FRO3 in planta* remains elusive. It is important to note that although mitochondrial homologs of FRO3 have been identified in other organisms (FRE5 in yeast) there is no evidence to date that these proteins function in mitochondrial iron metabolism (Jeong and Connolly, 2009).

Rice possesses only two FRO family members, *OsFRO1* and *OsFRO2*, neither of which has been shown to localize to mitochondria (Victoria Fde et al., 2012; Vigani, 2012). Thus, iron uptake by mitochondria of grass species such as rice may differ from non-grass species, like *Arabidopsis*. It is possible that iron uptake by rice mitochondria utilizes a non-reductive iron uptake pathway and/or the rice genome may encode other types of reductases capable of reducing iron. In the future, it will be critical to determine the redox state of iron transported across the outer and inner membranes of the mitochondria.

## FRATAXIN

Mitochondria are known to facilitate two major iron utilization pathways in the cell: heme synthesis and Fe–S cluster biogenesis. It has been suggested that the mitochondrial compartment contains micromolar concentrations of chelatable iron (Petrat et al., 2001). Maintaining this iron in a soluble and non-toxic form presents a challenge given the alkaline pH and the continuous production of ROS within the mitochondrial matrix under normal conditions (Park et al., 2002). Thus, the existence of mitochondrial iron chaperones and chelators was postulated (Flatmark and Romslo, 1975). Mitochondrial ferritin and FH have been implicated in iron storage and control of iron homeostasis in the mitochondrial matrix (Babcock et al., 1997; Corsi et al., 2002).

Frataxin is a conserved mitochondrial protein found in bacteria, yeast, mammals, and plants (Busi et al., 2006). FH was first identified in humans, where its deficiency was reported to cause an autosomal recessive cardio-neurodegenerative disease known as Friedreich’s ataxia (Campuzano et al., 1996). Functional studies in yeast revealed the role of FH (mYfh1p) in mitochondrial iron homeostasis (Babcock et al., 1997). Loss of yeast mYfh1p results in impaired iron export from the mitochondria. This is primarily due to the accumulation of iron as amorphous ferric phosphate nanoparticles, which are unavailable for physiological purposes (Lesuisse et al., 2003). Thus, although the *yfh1* mutant exhibits iron overload, it suffers from iron deficiency and thus upregulates the iron uptake machinery (Santos et al., 2010). Lack of FH also results in iron-induced oxidative damage of mtDNA and reduced activity of mitochondrial Fe–S cluster proteins which thus affects respiration (Foury, 1999). FH has been reported to bind to the ISC assembly complex suggesting its importance in Fe–S cluster biogenesis (Gerber et al., 2003). It directly interacts with a scaffold protein, Isu (iron–sulfur cluster U) in an iron-dependent manner and facilitates the transfer of iron to Isu during Fe–S cluster assembly. Because of the capacity of FH to bind iron and transfer it to Isu via a direct protein–protein interaction, FH is considered a mitochondrial iron chaperone (Philpott, 2012).

The first FH homolog identified in a photosynthetic organism was *Arabidopsis* AtFH (Busi et al., 2004); *AtFH* functionally complements the yeast FH mutant.* AtFH* is essential as loss-of-function mutants exhibit an embryo lethal phenotype (Vazzola et al., 2007). The knock-down mutant shows elevated levels of iron and ROS in the mitochondrial compartment (Martin et al., 2009). The oxidative stress observed in *atfh* mutants is accompanied by an increase in nitric oxide (NO) production. NO, a potent antioxidant, protects the cell by directly scavenging peroxide radicals (Beligni and Lamattina, 1999) and by inducing the expression of ferritin genes (*FER1* and *FER4*) to sequester free iron (Murgia et al., 2002; Martin et al., 2009).

Like its yeast ortholog, AtFH also functions as a mitochondrial iron chaperone. *atfh* mutants show reduced activity of two Fe–S cluster containing enzymes, mitochondrial aconitase and succinate dehydrogenase, while the activity of malate dehydrogenase (a non-Fe–S containing enzyme) is not altered. This indicates that FH likely plays a role in Fe–S cluster biogenesis and/or assembly of the Fe–S moiety with mitochondrial proteins in *Arabidopsis*. Indeed, it was shown that AtFH plays an instrumental role in Fe–S cluster biogenesis in plant mitochondria (Turowski et al., 2012). AtFH interacts with a cysteine desulfurase, AtNfs1m (which is known to supply S for the biogenesis of Fe–S clusters), and modulates its kinetic properties. AtNfs1m exhibits a 50-fold increase in its cysteine desulfurase activity in the presence of AtFH (Turowski et al., 2012). This interaction thus links the accumulation of iron (bound to FH) with Fe–S cluster production in a mitochondrion.

In animal systems, FH appears to be involved in the biogenesis of heme-containing proteins. FH was shown to interact with and deliver iron to ferrochelatase (FC) in the last step of heme synthesis in human mitochondria (Yoon and Cowan, 2004). Reduced FH expression in human cells results in reduced levels of heme-a and reduced cytochrome c oxidase activity (Napoli et al., 2007). In plants, however, there is no strong evidence to support the presence of FC in mitochondria. In fact, studies in various families of plants have clearly demonstrated the exclusive localization of FC to plastids (Cornah et al., 2002; Masuda et al., 2003). Therefore, it is possible that heme synthesis in plants occurs exclusively in plastids, some of which is then exported to the cytosol and mitochondria (van Lis et al., 2005; Tanaka and Tanaka, 2007; Mochizuki et al., 2010). Despite this, AtFH deficient plants show a decrease in total heme content, an altered expression of genes (*FC2*, *HEMA1*, *HEMA2*, *GSA1*, *GSA2*, *HEMB2*, *HEMF2*) which are involved in the heme biosynthetic pathway and a reduction in the activity of mitochondrial catalase, which is a heme-containing protein. Reduced catalase activity in *atfh* is rescued via supplementation with exogenous hemin (Maliandi et al., 2011). Taken together, these data indicate that in plants, FH plays important roles in protection against oxidative stress and in the biogenesis of Fe–S cluster and heme-containing proteins (Maliandi et al., 2011).

## MITOCHONDRIAL FERRITIN

Metal homeostasis in plants is accomplished via a set of elegantly regulated mechanisms that control various aspects of iron metabolism (including uptake, efflux, chelation, and storage). Ferritins are clearly essential to overall iron homeostasis as they function in iron sequestration and thus serve to prevent oxidative damage (Zhao et al., 2002; Arosio et al., 2009; Ravet et al., 2009). Ferritins are localized to both chloroplasts and mitochondria, two major sites for ROS production. Plant ferritins are conserved proteins that oligomerize to form a hollow sphere. They exhibit ferroxidase activity and oxidize Fe^2^^+^ and store it within the ferritin core in the form of hydrous ferric oxides along with phosphates (Arosio et al., 2009). Ferritin can accommodate 2,000–4,000 Fe^3^^+^ atoms per ferritin molecule (Carrondo, 2003). The molecular mechanism underlying the release of iron from ferritins is not very well understood. *In vitro* studies in animals suggest that release of Fe requires iron chelators or reducing agents. In contrast, *in vivo* studies in animals have demonstrated the release of Fe by proteolytic degradation of ferritin protein (Briat et al., 2010). To date, the process is not described in plant systems.

Plant ferritins are primarily localized to plastids, as opposed to animal ferritins which are usually cytoplasmic. Mitochondrial localization of ferritins was first reported in mammals (Levi et al., 2001). Subsequently, mitochondrial ferritins were also identified in plants (Zancani et al., 2004; Tarantino et al., 2010a). *Arabidopsis* possesses four ferritin (FER1–4) proteins, all of which are known to be localized to chloroplasts. Ferritin4 (AtFER4) is unique in that it contains dual targeting signals and is therefore found in mitochondria as well as chloroplasts (Tarantino et al., 2010a). This protein is detected in mitochondria in the aerial portion of plants only after exposure to excess iron. Although the *atfer4* mutant does not exhibit any severe phenotypes, callus cultures prepared from the *atfer4* mutant show reduced cell and vacuole size, damaged plasma membranes, accumulation of H_2_O_2_, higher cell death and reduction of O_2_ consumption, in addition to elevated cellular and mitochondrial iron concentrations (Tarantino et al., 2010b).

Loss of *AtFER4* also results in increased *FRO3* expression both in control as well as excess Fe conditions. This suggests that loss of *AtFER4* triggers sensing of mitochondrial iron deficiency despite the fact that mitochondrial iron levels are elevated in *atfer4*. This may also result in damage to electron transport chain components, which is consistent with the diminished O_2_ consumption rate of *atfer4* mutants. These observations indicate that although *AtFER4* is responsible for proper cellular iron homeostasis and subcellular iron trafficking, it is dispensable for protection against the oxidative stress in photosynthetic tissue (Tarantino et al., 2010a).

## CONCLUSION

Recent studies have begun to shed light on the machinery involved in mitochondrial iron uptake, storage, and trafficking/utilization. In particular, studies of mitochondrial iron transporters, chaperones, and storage proteins have set the stage for future investigations in this area (see **Figure 1**). Such studies will be critical to efforts to understand both organellar iron homeostasis and the mechanisms employed by plants to coordinate and prioritize Fe utilization by the various iron containing compartments of the cell. These studies will contribute to the development of a comprehensive understanding of iron homeostasis in plants, which should enable efforts to develop crop varieties with improved tolerance of growth on iron-limited soils and elevated levels of bioavailable iron in support of improved sustainability in agriculture and reductions in the incidence of iron deficiency in humans, respectively.

## Conflict of Interest Statement

The authors declare that the research was conducted in the absence of any commercial or financial relationships that could be construed as a potential conflict of interest.
